# Association between baseline circulating FGF21 levels and depressive symptoms at follow up in older adults: evidence from the FRASNET cohort

**DOI:** 10.3389/fpsyt.2026.1726636

**Published:** 2026-04-10

**Authors:** Sarah Damanti, Clara Sciorati, Amanda Avola, Rebecca De Lorenzo, Elena Brioni, Francesca Farina, Costanza Festorazzi, Martina Laffranchi, Martina Mallus, Marco Messina, Giulia Pata, Mariapia Ruggiero, Eleonora Senini, Simona Santoro, Laura Zagato, Lorena Citterio, Marco Simonini, Angelo A. Manfredi, Paolo Manunta, Chiara Lanzani, Patrizia Rovere-Querini

**Affiliations:** 1 Innate Immunity and Tissue Remodelling Unit, IRCCS Ospedale San Raffaele Scientific Institute, Milan, Italy; 2 Vita-Salute San Raffaele University, Milan, Italy; 3 Genomics of Renal Disease and Hypertension Unit, IRCCS San Raffaele Scientific Institute, Milan, Italy; 4 Nephrology and Dialysis Unit, IRCCS San Raffaele Scientific Institute, Milan, Italy

**Keywords:** biomarkers, depression, FGF21, geriatric depression scale, older adults

## Abstract

**Objectives:**

Fibroblast Growth Factor 21 (FGF21) is a stress-induced hepatokine involved in inflammation and neuroendocrine regulation, processes implicated in depression. We investigated the association between FGF21 and depressive symptoms in community-dwelling older adults.

**Methods:**

Data were obtained from 52 older individuals (median age 70; 61.5% women) within the FRASNET cohort who underwent longitudinal assessments (2017–2024). Depressive symptoms were evaluated using the 15-item Geriatric Depression Scale (GDS). Regression models adjusted for age, sex, and body mass index were used to assess associations between FGF21 and depressive symptoms.

**Results:**

Circulating FGF21 levels declined significantly over time (p = 0.03) but remained higher in individuals with depressive symptoms at both baseline and follow-up. Elevated baseline FGF21 predicted higher GDS scores at follow-up (adjusted B = 0.003, 95% CI 0.000–0.006, p = 0.049). Discriminatory performance for elevated depressive symptoms was modest (AUC = 0.66).

**Conclusions:**

Higher baseline FGF21 levels were associated with greater depressive symptom burden at follow-up. These findings should be considered preliminary and hypothesis-generating. Further studies using diagnostic outcomes and larger samples are warranted.

## Background

Depression in older adults is a complex and multifactorial condition with substantial consequences for physical health, cognitive trajectories, functional decline, and overall quality of life (1). Growing evidence indicates that late-life depressive symptomatology is closely intertwined with biological processes characteristic of aging, particularly metabolic dysregulation and chronic low-grade inflammation. These mechanisms are increasingly recognized as central contributors to the pathophysiology of neuropsychiatric conditions in older populations, extending beyond traditional neurotransmitter-centered models (1).

Recent studies have further emphasized the tight interconnection between metabolic disturbances and affective symptoms in aging. Notably, Mancinetti (2) reported a sex-specific association between insulin resistance and depressive symptoms in older adults, with vascular endothelial growth factor (VEGF) emerging as a potential mediator of this relationship. Such findings support the hypothesis that circulating metabolic and stress-related mediators may capture systemic processes relevant to mood regulation in later life.

Within this framework, Fibroblast Growth Factor 21 (FGF21) represents a biologically plausible candidate molecule.

FGF21 is a hepatokine primarily regulated by metabolic and nutritional stress signals, including mitochondrial dysfunction, energy imbalance, and cellular adaptive responses, rather than psychological stress. It plays a central role in glucose and lipid metabolism and has been implicated in systemic responses to metabolic challenge. Although experimental studies suggest that FGF21 signaling may exert neuroprotective and anti-inflammatory effects via activation of the β-Klotho/FGFR1c receptor complex (3), circulating FGF21 concentrations are generally interpreted as markers of metabolic stress and compensatory adaptation. Elevated FGF21 levels have been described in conditions characterized by insulin resistance, mitochondrial dysfunction, and chronic low-grade inflammation, suggesting that higher systemic concentrations may reflect an activated but potentially insufficient adaptive response.

From this perspective, increased FGF21 should not be considered inherently beneficial or detrimental. Instead, it may serve as an integrative signal of altered metabolic and inflammatory homeostasis—processes that are highly relevant in aging and have been implicated in depressive symptomatology. This interpretation is particularly pertinent to late-life depression, where multimorbidity, metabolic alterations, and systemic inflammation play a more prominent role than in younger individuals.

Empirical data linking FGF21 to depressive phenotypes remain limited and somewhat heterogeneous. Studies in younger populations have reported both elevated and reduced circulating FGF21 levels in association with depressive symptoms, suggesting that the biological significance of this molecule may be context- and age-dependent (4, 5). Evidence in older adults, however, is scarce.

Given the convergence between the biological functions of FGF21 and key mechanisms implicated in aging-related mood disturbances, the FRASNET cohort offers a valuable opportunity to explore the longitudinal relationship between circulating FGF21 levels and depressive symptoms in a well-characterized sample of community-dwelling older individuals.

## Methods

The Frailty and Sarcopenia Network (FRASNET) was a multicenter observational cohort study that included both healthy older adults living in the community and institutionalized elderly individuals. The study received ethical approval from the San Raffaele Scientific Institute (approval number: 24/INT/2017), and all participants provided written informed consent prior to enrollment. Recruitment took place between April 1, 2017, and October 16, 2020. Full inclusion and exclusion criteria have been described in earlier publications (6).

Between 2023 and 2024, participants were re-contacted via telephone. These follow-up calls aimed to confirm participants’ survival and evaluate their willingness to undergo in-person follow-up assessments. Eligible participants were invited to attend in-person follow-up visits. In addition to the geriatric evaluations conducted between 2017 and 2020, the new assessments offered a more comprehensive evaluation of functional ability, frailty, nutritional status, sarcopenia and mood (7).

Depressive symptoms were assessed using the 15-item Geriatric Depression Scale (GDS-15), a widely used screening tool designed to detect depressive symptomatology in older adults rather than to establish a clinical diagnosis of depression (8). Consistent with prior validation studies, a GDS-15 score ≥ 5 was used to indicate clinically relevant depressive symptoms according to screening criteria. We acknowledge that this threshold prioritizes sensitivity over specificity and therefore does not represent a diagnostic classification. Accordingly, all analyses and interpretations were conducted with reference to depressive symptoms identified by a screening instrument, not to clinically diagnosed depressive disorders.

The selection of aging-related biomarkers relevant to multimorbidity, frailty, and disability was carried out through a structured Delphi process involving multiple rounds of anonymous expert consultation to achieve consensus. The panel, composed of biologists and clinicians from several Italian universities, reviewed a range of candidate biomarkers (listed in **Supplementary Table 1**), ultimately selecting FGF21. FGF21 levels were measured using the ELLA™ automated immunoassay system (Bio-Techne, San Jose, CA, USA), based on blood samples collected and biobanked during the original FRASNET study (2017–2020) and during follow-up visits (2023–2024).

### Statistical analysis

Descriptive statistics were used to summarize baseline characteristics. Continuous variables were expressed as means with standard deviations (SD) when normally distributed, or as medians with interquartile ranges (IQR) when data were skewed. Categorical variables were presented as frequencies (N) and percentages (%). Changes in participant characteristics over time were analyzed using paired t-tests for normally distributed data and Wilcoxon signed-rank tests for non-normal data. Differences in categorical variables between baseline and follow-up were assessed using Chi-squared tests. To compare FGF21 levels by sex and affective status (depressed vs. non-depressed), the Mann–Whitney U test was used. Longitudinal changes in FGF21 were analyzed using the Wilcoxon signed-rank test. To further investigate the relationships between FGF21 and depressive symptoms linear regression analyses were conducted, adjusting for age, sex and body mass index (BMI). Given the low prevalence of participants exceeding the GDS-15 screening threshold, regression analyses were primarily designed to examine variability in continuous depressive symptom severity rather than a binary classification. Models based on the dichotomized outcome (GDS ≥ 5) were explored but yielded unstable estimates due to the limited number of events, and are therefore not presented to avoid potentially misleading inference.

To explore the discriminatory capacity of FGF21 relative to depressive symptom status defined by screening criteria, we performed a Receiver Operating Characteristic (ROC) curve analysis using the GDS-15 cut-off (≥5) as the reference classification. The ROC analysis therefore evaluates the ability of FGF21 to differentiate individuals with clinically relevant depressive symptoms according to the screening threshold, rather than clinically diagnosed depression. Optimal thresholds were identified using the Youden Index, which pinpoints the point on the ROC curve that offers the greatest difference between the true positive and false positive rates. All statistical analyses were performed with SPSS version 25.0 (SPSS Inc., Chicago, IL, USA).

## Results

**Figure 1:**
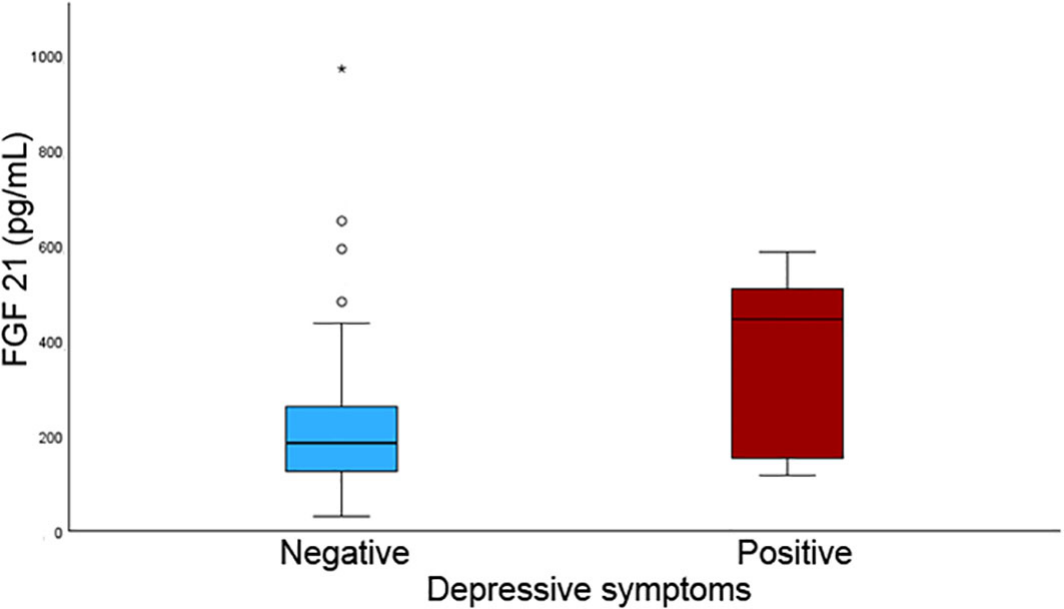
Distribution of baseline FGF21 concentrations according to depression screening status. Individuals screening positive for depressive symptoms exhibited higher FGF21 levels compared with those screening negative. Boxes represent interquartile ranges, central lines indicate medians, and whisker (*) denotes variability outside the upper and lower quartiles. Outliers are displayed as individual points.

**Figure 2:**
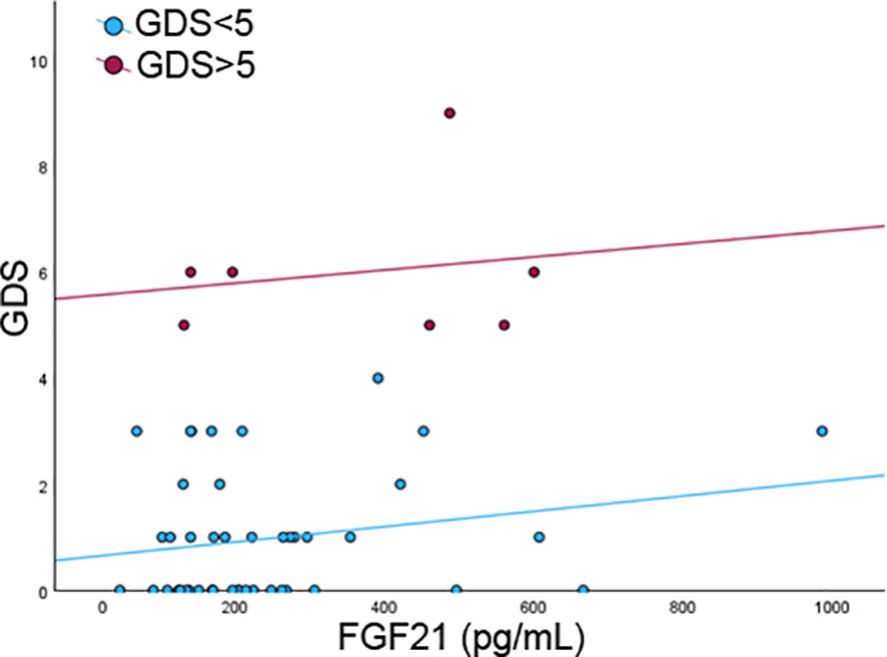
Association between baseline FGF21 concentrations and follow-up GDS-15 scores. The fitted regression lines illustrate the positive relationship between FGF21 levels and depressive symptom severity. Data points represent individual observations.

Among the 1,250 individuals originally enrolled in the FRASNET study, by November 2024, 228 completed follow-up visits. Biomarker analyses were restricted to a nested subgroup of 52 individuals with complete and good-quality stored plasma samples from both baseline and follow-up. At baseline, this subgroup had a median age of 70 years, and 61.5% were women. **Table 1** outlines the main demographic and clinical features of this cohort, along with changes observed between baseline and follow-up. Statistically significant differences emerged over time, including a slight but meaningful reduction in height (median 1.64 m at baseline vs. 1.625 m at follow-up, *p* < 0.001), an increase in waist circumference (93 cm to 98 cm, *p* < 0.001), higher scores on the Fatigue Severity Scale (24 vs. 33, *p* = 0.03), and an increase in the number of chronic medications reported (3 vs. 4, *p* < 0.001). Seven people (13.5%) had a GDS score ≥ 5 at follow-up visits. FGF21 levels declined significantly over time (p = 0.03), with median values dropping from 190 pg/mL (IQR 126.0 - 328.0) at baseline to 155 pg/mL (IQR 94.1 - 284) at follow-up. No significant differences in biomarker levels were found between the sexes (at baseline 195.0 pg/mL (IQR 127.3 - 275.0) in males and 186.0 pg/mL (IQR 126.0 - 349.8) in females, p =0.9; at follow-up 139.5 pg/mL (IQR 97.0 - 225.0) in males and 197.0 pg/mL (IQR 79.8 - 293.5) in females p = 0.95). FGF21 concentrations were higher in participants with depressive symptoms compared with those with GDS-15 scores < 5 at both baseline and follow-up. At baseline, median FGF21 levels were 446 (IQR 126–546) in individuals with depressive symptoms versus 186 (IQR 124.5–263.5) in those without. At follow-up, the corresponding values were 344.5 (IQR 148.5–615.8) and 133 (IQR 93.1–247.5), respectively. As shown in **Figure 1**, baseline FGF21 concentrations were higher among individuals screening positive for depressive symptoms at follow-up. The regression models were designed to examine the relationship between baseline FGF21 levels and subsequent depressive symptom severity at follow-up, rather than cross-sectional depressive status. Linear regression analyses indicated that baseline FGF21 concentrations were associated with follow-up GDS-15 scores, reflecting depressive symptom severity measured on a continuous scale (β 0.006, 95% C.I. 0.002 – 0.009, p = 0.001; standardized β = 0.305). These results were confirmed both at the age and sex adjusted analyses (β 0.003, 95% C.I. 0.00003 – 0.006, p = 0.048, standardized β = 0.278) and at the age, sex and BMI adjusted analyses (β 0.003, 95% C.I. 0.00001 – 0.006, p = 0.049, standardized β = 0.331). Furthermore, visual inspection of the regression plot (**Figure 2**) supported the linear regression findings, illustrating a positive association between baseline FGF21 concentrations and follow-up depressive symptom severity (GDS-15 scores).

**Table 1:** main characteristics of the 52 participants who underwent biomarkers assessment at baseline and during follow-up visits.

Parameters	2017-2020	2023-2024	p
Age	70.0 (IQR 68.0 - 73.0)	76 (IQR 74 - 78)	< 0.001
Females	32 (61.5%)	32 (61.5%)	N.A.
Height (cm)	1.64 (IQR 1.58 - 1.71)	1.62.5 (IQR 157.6 -171.0)	< 0.001
Weight	71.5 (IQR 65.3 - 77.8)	72.2 (IQR 66.1 - 77.8)	0.81
BMI	26.8 (IQR 24.8 - 29.7)	26.7 (IQR 25.1 - 30.2)	0.5
Waist circumference	93 (IQR 88 - 101)	98.0 (IQR 89.6 - 107.0)	< 0.001
SPPB total	10 (IQR 9 - 11)	11 (IQR 9 - 12)	0.08
FP	1 (IQR 1 - 1)	0 (IQR 0 - 1)	0.03
FI	0.10 (IQR 0.05 - 0.15)	0.13 (IQR 0.05 - 0.20)	0.22
PASE	105.5 (IQR 70.3 - 140.3)	109.0 (IQR 83 - 136.1)	0.88
MMSE	28 (IQR 27 - 30)	29 (IQR 28 - 30)	0.03
GDS 15 items	2 (IQR 0 - 3)	1 (IQR 0 - 3)	0.06
Fatigue severity scale	24.0 (IQR 17.5 - 31.0)	33 (IQR 20.25 - 41)	0.03
Number of chronic drugs	3 (IQR 1 - 4)	4 (IQR 3 - 6)	< 0.001

BMI, body mass index; FI, frailty index; FP, frailty phenotype, GDS, geriatric depression scale; MMSE, mini mental state examination; PASE, physical activity scale for the elderly.

**Figure 3:**
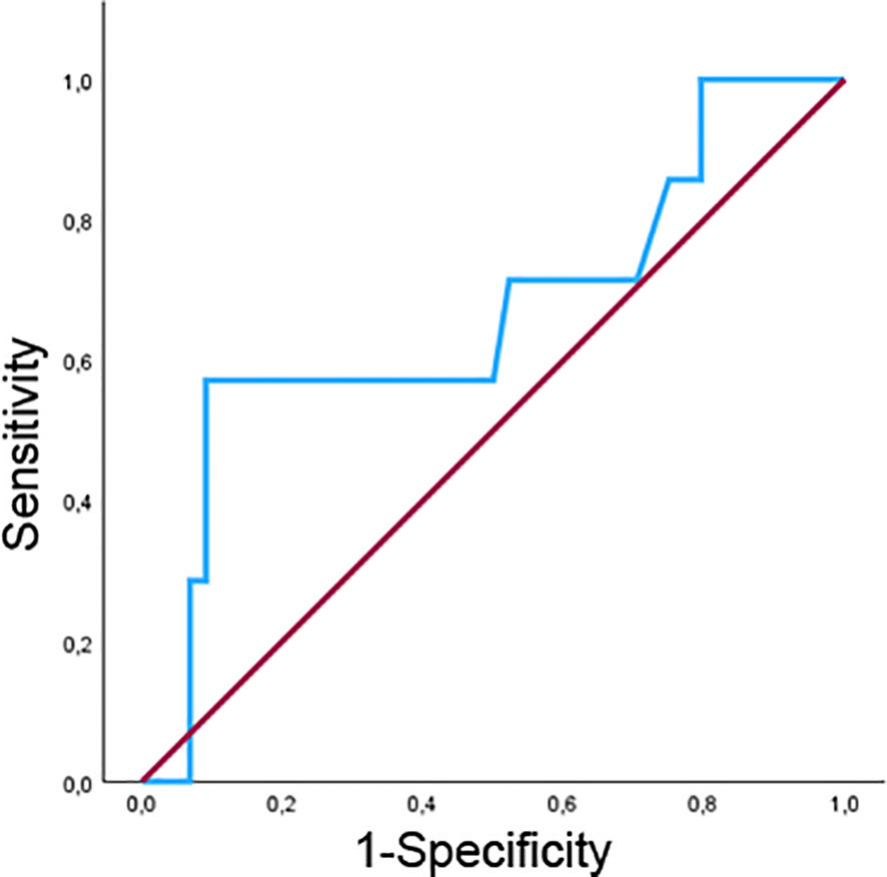
Receiver operating characteristic (ROC) curve evaluating the discriminative performance of baseline FGF21 concentrations for depression screening status. The curve illustrates sensitivity versus 1–specificity across thresholds. The reference diagonal represents chance-level discrimination.

ROC analysis (**Figure 3**) showed that baseline FGF21 concentrations provided modest discrimination for depressive symptom screening status (AUC = 0.66, 95% C.I. 0.42 – 0.91). The optimal threshold identified by the Youden Index was 442 pg/mL (Youden Index = 0.48).

## Discussion

In this prospective observational study of community-dwelling older adults, circulating FGF21 levels declined over time yet were higher among individuals exhibiting greater depressive symptom severity. Regression analyses indicated that baseline FGF21 concentrations were associated with follow-up GDS-15 scores, suggesting a relationship between this hepatokine and variability in depressive symptom burden. Importantly, these findings reflect associations with a continuous screening measure rather than diagnostic classification. The magnitude of the standardized coefficients suggests that the observed association is not only statistically significant but may also be clinically meaningful, particularly given the multifactorial biological underpinnings of late-life depressive symptoms.

The ROC analysis demonstrated modest discriminatory performance (AUC = 0.66), indicating limited capacity of FGF21 to differentiate individuals based on screening-defined depressive symptom status. This result is consistent with the exploratory nature of the study and underscores that the observed statistical associations should not be interpreted as evidence of diagnostic accuracy or clinical biomarker validity. Instead, the data suggest that FGF21 may capture biological variability accompanying depressive symptom expression within this cohort.

These findings align with emerging models of late-life depression that emphasize the contribution of metabolic and inflammatory dysregulation. FGF21 is primarily regulated by metabolic and nutritional stress signals and is widely regarded as a marker of systemic metabolic adaptation rather than a direct mediator of neuropsychiatric processes. Elevated circulating concentrations have been described in states characterized by mitochondrial dysfunction, insulin resistance, and chronic low-grade inflammation, all of which are mechanistically linked to aging and may plausibly relate to affective symptomatology. From this perspective, higher FGF21 levels may reflect underlying systemic stress or altered homeostasis rather than protective efficacy.

Prior literature examining FGF21 in relation to depressive phenotypes remains heterogeneous. Studies in younger populations have reported both increased and decreased circulating levels in association with depressive states, suggesting that the biological meaning of FGF21 alterations may be context- and age-dependent (4, 5). Experimental evidence indicating neuroprotective and anti-inflammatory effects of FGF21 (9) signaling does not necessarily contradict the present findings, as elevated systemic levels may represent compensatory activation or reduced signaling efficiency, phenomena observed in several metabolic regulatory pathways. Similarly, discrepancies with cerebrospinal fluid studies (10) highlight the complexity of peripheral versus central FGF21 dynamics.

Taken together, our results support the hypothesis that FGF21 may represent a peripheral correlate of biological processes associated with depressive symptom variability in older adults

Strengths of this study include the use of a well-characterized cohort of older adults, standardized biomarker assess measurements, and adjustment for key confounders. Limitations include the modest sample size of the biomarker subcohort, which reflects the availability of stored plasma samples, as well as the single-center design, both of which may restrict generalizability. Moreover, the ROC analysis was performed using a screening-based classification derived from the GDS-15 cut-off rather than a diagnostic assessment of depressive disorder. Therefore, the AUC estimates reflect the ability of FGF21 to discriminate individuals with elevated depressive symptoms according to screening criteria, not clinically established depression. Given the limited specificity of the scale threshold, these results should not be interpreted as evidence of diagnostic biomarker performance. Another limitation of our study is the lack of data on insulin resistance. Since FGF21 levels are known to be influenced by metabolic status and may be affected by insulin resistance and related treatments, we cannot exclude that these factors may have contributed to the observed associations. Accordingly, our findings should be considered preliminary and warrant confirmation in larger, more diverse populations.

## Conclusions

Circulating FGF21 levels were associated with depressive symptom severity in this cohort of older adults. These findings suggest that FGF21 may reflect biological processes relevant to late-life affective symptomatology rather than serving as a diagnostic marker. Given the screening-based outcome and modest sample size, the results should be considered preliminary and hypothesis-generating. Further investigation in larger cohorts incorporating diagnostic assessments is warranted.

## Data Availability

The raw data supporting the conclusions of this article will be made available by the authors, without undue reservation.
