# Supplementary material for: Association between baseline circulating FGF21 levels and depressive symptoms at follow up in older adults: evidence from the FRASNET cohort
Source: Front Psychiatry. 2026 Apr 10;17:1726636. doi: 10.3389/fpsyt.2026.1726636 (PMC13106514; doi:10.3389/fpsyt.2026.1726636)
Supplement: Supplementary file 1 [file Table1.docx]

**Supplementary Table 1**: Proteins considered as potential biomarkers

| GDF15 |
| --- |
| FGF21 |
| NfL |
| sST2 |
| CXCL9 |
| IL-6 |
| IL-6Ralpha |
| gp130 |
| BDNF |
| sRAGE |
| ACTIVIN |
| OSTEOPONTIN |
| FOLLISTATIN |
